# A double-blinded randomised controlled study to investigate the effect of intraperitoneal levobupivacaine on post laparoscopic pain

**Published:** 2020-10-08

**Authors:** TK Cunningham, H Draper, H Bexhell, V Allgar, J Allen, D Mikl, K Phillips

**Affiliations:** Department of Obstetrics & Gynaecology, Hull University Teaching Hospitals NHS Trust, Hull, United Kingdom, HU32JZ; Centre for Health and Population Sciences, Hull York Medical School, Hull, United Kingdom, HU6 7RX

**Keywords:** Bupivacaine, intraperitoneal instillation, postoperative analgesia, pain, laparoscopy

## Abstract

**Background:**

Laparoscopic surgery is the cornerstone of modern gynaecological surgery, with shorter hospital stays and a quicker return to normal activities. However postoperative pain remains problematic. No strategy to reduce phrenic nerve irritation, including heating or humidifying the insufflating gas, alternatives to CO_2_, and intraperitoneal analgesics, has shown superiority.

**Methods:**

100 women undergoing laparoscopic surgery were randomly allocated, having either 40ml of 0.25% levobupivacaine or 40ml 0.9% sodium chloride solution administered into the peritoneal cavity following surgery. The patients and the main researcher were blinded. All women received standardised anaesthetic and laparoscopic technique, and postoperative pain control including nursing position and nature of analgesia. Postoperative pain was assessed 3 hours, 8 hours, day 1 and day 4/5 postoperatively.

**Results:**

100 patients were recruited undergoing surgery for benign causes aged 19-73(mean 40.3±13). There was no difference between the groups for age(p=0.64) or length of operation(p=0.56). There were no adverse events related to use of intraperitoneal instillation. There was a significant reduction in shoulder-tip pain scores in the levobupivacaine group at 3 hours(p=0.04). Furthermore, there was a significant reduction in wound-pain scores in the levobupivacaine group at 8hrs(p=0.04) and at day 4(p=0.04). No difference was found in pelvic pain between the two groups. No significant difference was found in the use of post-operative analgesia.

**Conclusions:**

Intraperitoneal instillation of 40ml of levobupivacaine has some benefit in reducing postoperative pain and need for analgesia in the initial hours following gynaecological surgery. However, further well-designed randomised control trials are required to decide the optimum route and concentration of administering local anaesthetic.

## Introduction

Laparoscopic surgery has transformed modern gynaecological surgery. Its many advantages include shorter hospital stay, reduced blood loss, smaller incisions, and overall reduced morbidity for the patient (Wills and Hunt, 2000). However, many patients suffer from severe shoulder tip and abdominal pain in the early postoperative period that can delay hospital discharge and often require strong analgesia (Ng et al., 2004; Elhakim et al., 2000).

The cause of the pain is multifactorial; visceral pain due to stretching of the abdominal cavity, and peritoneal irritation due to entrapment of dissolved CO_2_. Shoulder tip pain arises due to phrenic nerve irritation, again as a result of CO_2_ trapped under the diaphragm (Wills and Hunt, 2000). Less frequently parietal pain occurs at the surgical incision site (Hernández-Palazón et al., 2003). Following laparoscopic surgery patients often complain of severe pain within the first 24 hours (Bisgaard et al., 2001). Different methods have been used in an attempt to reduce this immediate post-operative pain, which include varying analgesic regimes to the type of gas used to insufflate the abdomen.

Parenteral (opioid) narcotics are effective medications but increase morbidity and delay discharge (Tan et al., 2014). Non-narcotic medications are preferred as the patient can take them home after day surgery. Opioids have multiple adverse effects that include nausea, vomiting and sedative effects, whilst non-steroidal anti-inflammatory drugs (NSAIDs) can cause gastric mucosal irritation and impaired platelet and renal function, particularly in starved patients prior to general anaesthesia (Tan et al., 2014).

It has already been demonstrated that not only is the intensity of the pain proportional to the size of the residual gas bubble beneath the diaphragm (Jackson et al., 1996), but that attempting to expel all the gas and nursing the patient head down immediately postoperatively are successful techniques to reduce pain (Fredman et al., 1994). Carbon dioxide is the insufflation gas of choice because of its high solubility coefficient, and the fact that it is non-combustible. It had been suggested that carbonic acid resulting from the exposure of CO_2_ to peritoneal fluid might lead to acidic irritation of the peritoneum, however, NO_2_ did not reduce pain in a randomised controlled trial (Lipscomb et al., 1994) and Helium did not reduce the duration of postoperative stay (Korell et al., 1996). Heating the CO_2_ prior to insufflation was advocated as a way of reducing postoperative pain. However, there is disagreement between 2 large prospective randomised trials as to whether heating the insufflating gas causes a reduction (Korell et al., 1996) or an increase (Slim et al., 1999) in postoperative pain.

Intraperitoneal instillation of local anaesthetic levobupivacaine has been used to help minimise post-operative pain following laparoscopic surgery. Levobupivacaine is a long acting local anaesthetic with less cardiovascular toxicity (Knudsen et al., 1997). It has previously been demonstrated that intraperitoneal instillation can improve postoperative pain relief after laparoscopy but is only effective for a short period of time and needs to be specifically applied to the painful area (Simpson et al., 1999). A review of 13 trials of intraperitoneal levobupivacaine (50 to 200mg in volumes of 10 to 100mls) showed that significant pain reduction only occurred in 7 of the 13 studies, and that the duration of the analgesia when present was only 2 hours (Møiniche et al., 2000). The magnitude and duration of any analgesic effect is dose related and hence limited by the potential systemic toxic effects (Ng et al., 2002). Whilst there is a growing evidence base particularly in laparoscopic cholecystectomy where post-operative pain scores are noted to be significantly lower (Hernández-Palazón et al., 2003), there are few studies addressing this question in gynaecological laparoscopy.

The aim of this randomised double-blind study was to compare post-laparoscopic pain in women treated with intraperitoneal instillation of levobupivacaine compared with intraperitoneal instillation of 0.9% sodium chloride, and to assess whether intraoperative instillation of levobupivacaine reduces the need for postoperative narcotic analgesia.

## Materials and methods

A single-centre prospective randomised double- blind placebo-controlled study was conducted. Women were recruited from Hull University Teaching Hospitals NHS Trust in the UK and provided written consent following ethical approval (REC 09/H0106/47). The trial was registered on the European Union Clinical Trials Register (EudraCT No. 2009011207-23). The women had an American Society of Anesthesiologists (ASA) anaesthesia risk classification status 1-2 and were scheduled to have laparoscopic surgery under the same gynaecological surgeon. Exclusion criteria were women with a BMI>35 due to increased surgical risks and complexity, those who weighed less than 50kg due to risk of local anaesthetic toxicity, those with an ASA >2, or those with a known previous adverse reaction or any contraindication to levobupivacaine.

The patients were informed about the numerical pain rating scale (NRS) with 0 being no pain and 10 being the worst possible pain. This allowed answers to be conveyed over the telephone following discharge. A score would be requested for pain for the abdominal wound sites, pelvis, and shoulder tip.

The patients were randomly assigned into two groups by computer randomisation. Group 1 (n =50) would receive 40 ml of intraperitoneal 0.25% levobupivacaine, and Group 2 (n = 50) would receive 40ml of intraperitoneal sodium chloride 0.9%. Due to the lack of conformity in the literature, the dose of levobupivacaine was decided due to the clinical experience of the team and the maximum non-toxic dose of the minimum weight patient included in the study. The study was double blinded; the patients, surgical and anaesthetic teams were blinded to the medication used. The packets containing either 0.9% saline or 0.25% levobupivacaine solutions were prepared within pharmacy by computer randomisation code, blinded, and opened by the anaesthetists after induction of anaesthesia.

After intravenous cannulation, all patients received intravenous antiemetic prophylaxis consisting of Atropine 400-600ug, Metoclopramide 10mg, Ondansetron 8mg and Dexamethasone 6.6mg followed by 100ug of Fentanyl. Following pre- oxygenation, general anaesthesia was induced with 2-3 mg/kg of Propofol and 0.5mg/kg of Atracurium. All patients were mechanically ventilated with 50% mixture of O_2_ +N_2_O and Sevoflurane. Standard ventilator settings were: PCV-VG mode, Vt of 500mls, frequency 12/min, inspiratory to expiratory ratio of 50% and maximum inspiratory pressures were limited to 30cm H_2_O. The aim was to achieve mild hypocapnia at 4.5-4.8% end expiratory CO_2_ and haemoglobin saturation with O_2_ of 96-98%. Prior to the insertion of umbilical port every patient received 10 mg of Morphine intravenously (iv). All patients underwent their planned gynaecological surgery in a Lloyd Davis position with the table in a 20 degree Trendelenburg tilt.

Laparoscopic technique was standardised as far as practicable. The same lead surgeon performed a closed umbilical laparoscopic entry with a Verres needle, initial 20mmHg pressures to facilitate a 10mm umbilical and two 5mm lateral port insertion followed by operating pressure of 12mmHg.

During surgery, heart rate, non-invasive arterial blood pressure, oxygen saturations, end-tidal CO_2_ values and the total requirement of anaesthesia was recorded at 5-minute intervals. Following completion of the surgery Group I was instilled with 40ml of 0.25% levobupivacaine and Group II 40ml of normal saline via the lateral port and directed under vision towards the pelvis. The CO_2_ was then expelled from the abdomen through the umbilical trochar and the tilt was corrected. The incision sites were closed with a 2/o caprosyn suture.

Once the patient was taken into the postoperative recovery area this was defined as hour 0, with the first pain score being taken 3 hours following this time. Pain scores were assessed via questionnaire at 3 hours, 8 hours, 24 hours, and 96-120 hours post- operatively. Routine safety monitoring took place in the recovery area, including blood pressure, pulse and pulse oximetry, urine output, vaginal loss, and the patients were all nursed in the same position. Monitoring continued postoperatively at standard intervals until discharge. Standard postoperative pain control had been agreed, with a record of all postoperative analgesia administered whilst in the hospital and taken after discharge until the final pain score at day 4/5. The patients were asked to give a pain score for the pelvis, shoulder tip and wound site at 3, 8, 24 hours and day 4/5 following the surgery.

Using the data from a previous study by Sharma et al. (2014) a power calculation could be made. They found that the SD for pain score was 1.4, so based on this a sample of 100 patients (50 per group) would allow us to detect a difference in pain scores between groups of at least 0.8 on the NRS, based on 80% power and 5% significance. There is no accepted standard in the literature as to what represents a clinically significant reduction in pain score (Olsen et al. 2017).

## Statistical Analysis

Descriptive data is presented as mean ± SD for continuous data and n (%) for categorical data. T-tests were used to compare continuous data, Mann Whitney for ordinal data (e.g. pain scores) and Chi- square tests for categorical data. For the primary outcome measure (NRS), Mann Whitney tests were used to compare pain scores between groups at each time point. For the primary endpoint (8 hours), ANCOVA was used to compare pain scores between groups, whilst adjusting for baseline scores. In addition, potentially confounding variables were included (age and operative time).

## Results

100 patients were included in the study; 50 received 40mls intraperitoneal 0.9% saline and 50 received 40mls 0.25% intraperitoneal levobupivacaine. Patients were aged 19-73 (mean 40.3 ± 13). There was no significant difference between the groups for either age (MW p=0.64) or operative time (MW p=0.56) (Table I). All patients were undergoing surgery for benign causes. Of the 50 women in each group, 25 were undergoing laparoscopic treatment for endometriosis, all of whom were found to have stage 3 or 4 endometriosis at the time of surgery. All patients received the standardised anaesthetic and surgical techniques described above. There were no significant surgical intra-operative complications and the total estimated blood loss was <100mls for all patients. There was no difference between the groups for adverse events (p>0.1). There were 3 adverse events in total. One in the 0.25% bupivacaine group, where the patient reported pain secondary to pre-existing irritable bowel syndrome. One patient experienced itching at the cannulation site following administration of IV morphine, and one patient aspirated under anaesthesia (prior to administration of intra-peritoneal 0.9% saline) requiring a prolonged hospital admission.

**Table I:** Mean demographics for age, operation and mean operating time.

		Total	0.9% Saline	0.25% Levobupivacaine
Mean Age (years) ± SD (range)		40 ± 13 (19-73)	40 ± 12 (19-71)	41 ± 13 (22-73)
Mean Operating Time (minutes) ± SD (range)		66 ± 30 (25-230)	69 ± 36 (25-230)	62 ± 23 (30-145)
Indication for surgery		100	50	50
	Hysterectomy +/- salpingo-oophrectomy	25	14	11
	Bilateral salpingo-oophrectomy	8	2	6
	Unilateral salpingo-oophrectomy	7	3	4
	Treatment to endometriosis	50	25	25
	Laparoscopic sacrocolpopexy/hysteropexy	10	6	4

**Table II:** Descriptive summary of the pain scores at each time point by group.

	Group									
	0.9% sodium chloride					0.25% levobupivacaine				
	Mean	SD	n	Lower quartile	Upper quartile	Mean	SD	n	Lower quartile	Upper quartile
Wound										
3 hour	3.6	3.0	49	1	6	2.4	2.2	49	1	4
8 hour	4.1	3.0	44	2	7	2.9	2.4	47	0	5
1 day	3.6	2.5	49	2	6	3.1	2.5	49	1	5
4 day	3.1	2.1	51	1	5	2.2	1.7	50	0	4
Pelvis										
3 hour	3.1	2.7	49	1	5	3.0	2.5	49	1	5
8 hour	3.6	2.9	44	1	7	3.0	2.3	47	1	4
1 day	3.2	2.4	49	1	5	3.5	2.7	49	1	6
4 day	2.5	2.4	51	0	5	2.0	2	50	0	3
Shoulder tip										
3 hour	1.9	2.8	48	0	3	0.8	1.6	49	0	1
8 hour	2.8	3.1	44	0	6	1.5	2.4	47	0	3
1 day	2.1	3.1	49	0	5	1.6	2.4	49	0	3
4 day	1.1	2.1	51	0	2	0.8	1.9	50	0	0

**Figure 1:**
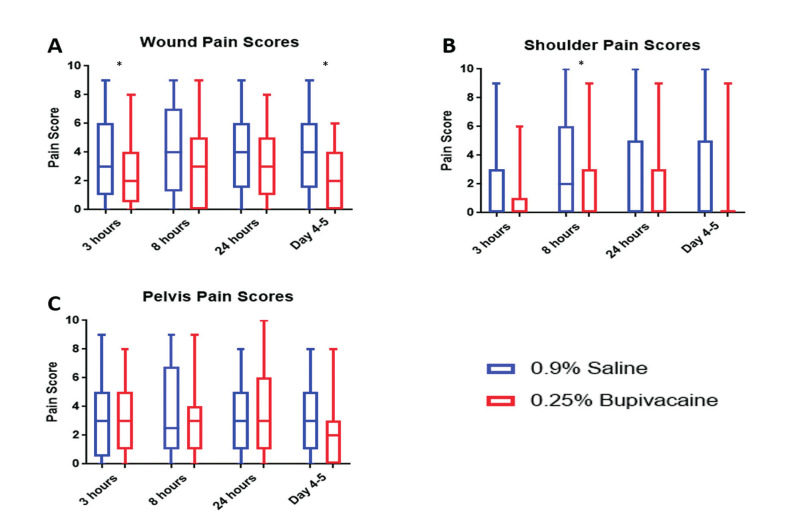
— Box plots showing mean pain scores at 3, 8, 24 and 96-120 hours post-surgery for; A) wound, B) shoulder and C) pelvis pain. * denotes p<0.05

There was a significant difference in the wound pain scores, with the 0.25% levobupivacaine group having lower pain scores at 8hrs (MW: p=0.04) and at day 4 (MW: p=0.04), compared to 0.9% sodium chloride group (Figure 1), although this lost significance at 3hrs (MW: p=0.06) and day 1 (MW: p=0.247).

There was a significant difference in shoulder tip pain scores, with the 0.25% levobupivacaine group having lower pain scores compared to the 0.9% saline group at 3 hours (MW p=0.04), although significance was lost at 8hrs (MW p=0.06) and not regained.

No significant difference was found for pelvic pain at any timepoint. An ANCOVA test was used to compare reported pain at 8 hours when adjusted for the pain score at 3 hours thereby adjusting for baseline pain. No significant difference between the groups was seen for pelvic (MW p=0.20), shoulder tip (MW p=0.19) or wound pain (MW p=0.18). Similarly, no difference between groups was seen when adjusted for age or length of operation.

**Figure 2:**
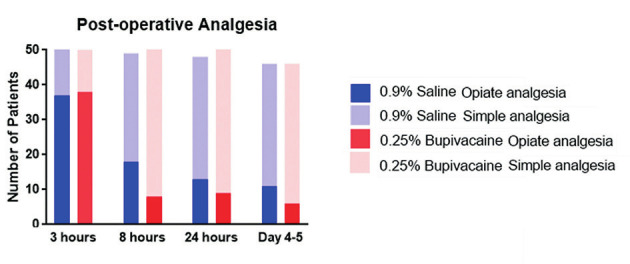
— Analgesia requirement at 3, 8, 24 and 96-120 hours postoperatively for the bupivacaine and normal saline groups.

The requirement for post-operative analgesia was also assessed (Figure 2). There was no significant difference found between the groups in the need for post-operative analgesia in terms of type of analgesia. When assessing the descriptive statistics at 8 hours a higher proportion used oral opioid analgesics in the 0.9% sodium chloride group (24%) when compared to 0.25% levobupivacaine group (6%) although this did not reach statistical significance (MW p=0.06). There was no statistical difference found between the groups for frequency of analgesia required; 12% required analgesia more than twice daily in the bupivacaine group at 8 hours in comparison to 19% in the saline group. In the saline group 19% of patients required analgesia four times daily at 24 hours compared to 10% in the bupivacaine group.

A subgroup analysis was performed on patients who had undergone laparoscopy for treatment of endometriosis (n=50), 25 in the 0.9% saline group and 25 in the 0.25% levobupivacaine group, all of whom had stage 3 or 4 disease. There was a significant reduction in wound pain at 3 hours (MW p=0.015), 8 hours (MW p=0.016) and 4 days (MW p=0.013) as well as shoulder pain at 3 hours (MW p=0.04) and 4 days (MW p=0.04) in the 0.25% levobupivacaine group.

## Discussion

The advantages of laparoscopic surgery for many gynaecological procedures is the quick recovery time and reduced hospital stay. However early postoperative pain after laparoscopy is a result of several factors including trauma to the abdominal wall, abdominal distension, and peritoneal irritation secondary to the pneumo-peritoneum using CO_2_ (Kaloo et al., 2019). Visceral and shoulder tip pain tend to be the most problematic, with visceral pain having its maximum impact during the first few hours after surgery exacerbated by mobilisation and respiration. This visceral and shoulder tip pain can be blocked by intraperitoneal infiltration (Narchi et al., 1991). Our study has demonstrated that intraperitoneal 0.25% levobupivacaine is a safe and easy intervention for post laparoscopic surgery pain reduction. There was a significant reduction in the shoulder tip pain in the levobupivacaine group at 3 hours (p=0.36), with a trend of this continuing to 8 hours that was just outside statistical significance (p=0.06). Whilst Narchi et al. (1991) and Goldstein et al. (2000) found an ongoing analgesic benefit, the loss of efficacy would be in keeping with the half-life of the levobupivacaine, with no significant difference noted at the other recorded time intervals. In fact, when a subgroup analysis was performed, those being treated for stage 3 or 4 endometriosis reported a more prolonged action with a significant reduction in wound pain at 3 hours (p=0.015), 8 hours (p=0.016) and 4 days (p=0.013) as well as shoulder pain at 3 hours (p=0.04) and 4 days (p=0.04).

Previous studies have demonstrated that instillation of 100mg intraperitoneal levobupivacaine did not cause toxicity and provided good pain relief. Narchi et al. (1991) demonstrated intraperitoneal infiltration of 40ml of 0.25% levobupivacaine provides effective analgesia with plasma levels below toxic level of 3ug/ml. . Levobupivacaine was used because of its prolonged duration of action with a half-life of 5 -16 hours. A double blinded RCT by Mahotra et al. (2007) demonstrated that instilling 100mg of levobupivacaine provided a longer analgesic effect compared to 50mg of levobupivacaine 8 hours vs. 4-6hours. However, studies by Shaw et al. (2001) and Keita et al. (2003) found no benefit of intraperitoneal levobupivacaine following laparoscopic gynaecological surgery.

The local anaesthetic agent and preparation of choice has not been well investigated yet. Goldstein et al. (2000) compared 100mg of levobupivacaine, 150mg of ropivacaine with normal saline as placebo and found that both pain scores and analgesia requirement were greater for levobupivacaine compared to ropivacaine. However, Sharma et al. (2014) performed an RCT on intraperitoneal and periportal levobupivacaine vs. intraperitoneal and periportal ropivacaine and found both to be effective at reducing pain in patients undergoing laparoscopic cholecystectomy with no significant difference between the groups. This is despite ropivacaine having 60% the potency of levobupivacaine. Whilst there are design limitations in many of these studies, this heterogeneity suggests a significant role for confounding factors.

The reduction in pain scores seen initially in both wound and shoulder-tip pain scores did not translate into a reduction in the analgesic requirements. Whilst the levobupivacaine group demonstrated a trend towards a reduction in the need for opiate analgesia, it did not reach statistical significance (p=0.06). This is likely due to the residual pelvic pain which was not altered by the intervention. However, the need for analgesia is a complex interaction of experienced pain, patient expectations, and nursing protocols. We undertook several other measures to reduce the post-operative need for analgesia including the use of intra-operative fentanyl and careful expulsion of residual CO_2_ . The existing literature shows no consensus in results with Mahotra et al. (2006) demonstrating that women receiving intraperitoneal levobupivacaine required less analgesia compared to controls following gynaecological surgery. However, Chundrigar et al. (1993) reported a similar pattern of results with a reduction in postoperative pain in the initial few hours when 0.25% of levobupivacaine was given intraperitoneally after laparoscopic cholecystectomy compared to controls, but these patients still required the same analgesic requirement over the first 24 hours.

Andrews et al. (2014) compared a continuous infusion of local anaesthetic for 48 hours post- operatively and assessed the patient-controlled analgesia morphine requirement. They found no opioid sparing effects when compared to 0.9% saline or reduction in hospital stay. However, this study was limited by a level of bias introduced by a relatively small sample size and a significant proportion of women being intolerant to NSAIDs in the treatment arm.

An RCT on women undergoing laparoscopy for gynaecology procedures by Butala et al. (2013) instilled intraperitoneal levobupivacaine with morphine, which resulted in significantly prolonged time before the first rescue analgesia and reduced the total consumption of analgesia in 24 hours postoperatively. Colbert et al. (2000) observed that combining intraperitoneal levobupivacaine with meperidine gave a greater analgesic effect compared to intraperitoneal levobupivacaine and intramuscular meperidine following laparoscopic tubal ligation. Karaman et al. (2014) compared intraperitoneal levobupivacaine vs. periportal levobupivacaine plus intraperitoneal levobupivacaine vs. control. There was no significant difference in pain scores between the levobupivacaine groups although they both reported significantly less pain than the controls. However, the periportal levobupivacaine group required less postoperative analgesia compared to intraperitoneal instillation only.

There was no difference between the groups for adverse events (p>0.1). There were 3 adverse events in total. One in the 0.25% levobupivacaine group, where the patient reported pain secondary to pre-existing irritable bowel syndrome. One patient experienced itching at the cannulation site following administration of IV morphine, and one patient aspirated under anaesthesia (prior to administration of intra-peritoneal 0.9% saline) requiring a prolonged hospital admission. There were no significant surgical intra-operative complications.

A recent Cochrane review has assessed the use of wound infiltration of local anaesthetic in post-operative pain following laparoscopic cholecystectomy. They conclude that whilst serious adverse events are rare, the quality of evidence for reduction of pain is low and the clinical importance is small (Loizides et al., 2014). Furthermore, the use of local anaesthetic infiltration at the port site alone has no impact on the troubling symptom of shoulder-tip pain. The use of intraperitoneal 0.25% levobupivacaine is a safe and easy technique with no added surgical time which can be readily adopted in a general gynaecological surgery setting. Other techniques which have been investigated including transversus abdominis plane (TAP) block or continuous infusion all have an associated cost of either equipment or training.

In conclusion, intraperitoneal instillation of 40ml of 0.25% levobupivacaine has some benefit in reducing postoperative pain in the initial few hours following gynaecological surgery. It is a safe and easy to perform technique with, no additional need for equipment, time or training, and may have some reduction in need for analgesia.
